# Addressing recall bias in (post-)conflict data collection and analysis: lessons from a large-scale health survey in Colombia

**DOI:** 10.1186/s13031-022-00446-0

**Published:** 2022-04-08

**Authors:** Rodrigo Moreno-Serra, Misael Anaya-Montes, Sebastián León-Giraldo, Oscar Bernal

**Affiliations:** 1 Centre for Health Economics, University of York, Alcuin A Block, Heslington, YO10 5DD UK; 2 Alberto Lleras Camargo School of Government, Universidad de Los Andes, Colombia, Carrera 1° N° 19-27, Bloque AU, piso 3, Bogotá, Colombia

**Keywords:** Conflict, Health, Household surveys, Recall bias, Causal effects, Survey data, Flashbulb test, Test-retest

## Abstract

**Background:**

Much applied research on the consequences of conflicts for health suffers from data limitations, particularly the absence of longitudinal data spanning pre-, during- and post-conflict periods for affected individuals. Such limitations often hinder reliable measurement of the causal effects of conflict and their pathways, hampering also the design of effective post-conflict health policies. Researchers have sought to overcome these data limitations by conducting ex-post surveys, asking participants to recall their health and living standards before (or during) conflict. These questions may introduce important analytical biases due to recall error and misreporting.

**Methods:**

We investigate how to implement ex-post health surveys that collect recall data, for conflict-affected populations, which is reliable for empirical analysis via standard quantitative methods. We propose two complementary strategies based on methods developed in the psychology and psychometric literatures—the Flashbulb and test-retest approaches—to identify and address recall bias in ex-post health survey data. We apply these strategies to the case study of a large-scale health survey which we implemented in Colombia in the post-peace agreement period, but that included recall questions referring to the conflict period.

**Results:**

We demonstrate how adapted versions of the Flashbulb and test-retest strategies can be used to test for recall bias in (post-)conflict survey responses. We also show how these test strategies can be incorporated into post-conflict health surveys in their design phase, accompanied by further ex-ante mitigation strategies for recall bias, to increase the reliability of survey data analysis—including by identifying the survey modules, and sub-populations, for which empirical analysis is likely to yield more reliable causal inference about the health consequences of conflict.

**Conclusions:**

Our study makes a novel contribution to the field of applied health research in humanitarian settings, by providing practical methodological guidance for the implementation of data collection efforts in humanitarian contexts where recall information, collected from primary surveys, is required to allow assessments of changes in health and wellbeing. Key lessons include the importance of embedding appropriate strategies to test and address recall bias into the design of any relevant data collection tools in post-conflict or humanitarian contexts.

**Supplementary Information:**

The online version contains supplementary material available at 10.1186/s13031-022-00446-0.

## Background

Civil conflicts have become more common and more physically destructive since the mid-20th century, affecting poorer countries disproportionately, with devastating consequences for health [1, 13, 14]. Beyond the immediate effects through casualties, population health is affected in the longer term by destruction of economic assets, damage or lack of access to public infrastructure, and population displacements. The health consequences of conflicts are likely to aggravate poverty and inequalities through, among others, reduced ability to work, depletion of assets and savings to cope with health losses, and financially catastrophic healthcare payments [15].

Assessing the dynamics of health and living standards during and after conflict is critical to the development of public policies that are effective in promoting the health and wellbeing of conflict-affected populations [16]. Nevertheless, much of the existing research on the consequences of civil conflicts for population health—and for development, more generally—has been constrained by severe data availability limitations, in particular the absence of longitudinal data for conflict-affected individuals spanning pre-, during- and post-conflict periods [7]. These data limitations hamper not only the identification of trends in health and wellbeing in the periods during and after conflict violence, but also the reliable quantification of (1) the *causal effects* of conflict exposure on post-conflict population outcomes (net of the influence of other socio-economic determinants of these outcomes), as well as of (2) the causal pathways for these effects. Often, these data shortcomings have resulted in studies drawing conclusions from methods that are inherently flawed to support reliable inference (e.g. simple before-after comparisons of health outcomes for conflict-affected individuals, with no suitable comparison group), being therefore unable to offer robust guidance for the implementation of post-conflict policies that can effectively target those groups that are most vulnerable to the damaging consequences of conflict violence [1, 14].

Violence and security concerns are common obstacles for data collection efforts in times of live confrontations, and even in the aftermath of a formal cessation of hostilities. Researchers have usually sought to overcome the impossibility of collecting household or individual baseline data during conflict periods by conducting surveys after the events, asking participants to recall their health and living standards before the onset of, or during, conflict [7]. Although potentially useful, these questions may introduce important biases due to recall error, which have been studied in the psychometric literature [3, 23–25]. Whilst some of these concerns can be considered relatively standard for any analyses relying on recall survey questions, other issues pertain more specifically to the case of health research undertaken in conflict or humanitarian settings, where trauma may further influence recall.

First, it is well established that recall may change depending on the language that is used in questionnaires, i.e. how questions are framed or phrased [18]. Second, recall ability decreases over time, so long recall periods may potentially lead to little or no memory about specific events, which could explain why some people fail to report noteworthy past events, for example hospitalisations [17, 23]. Third, memory can fail, in the sense that events that an individual did not truly experience may be involuntarily, erroneously “retrieved” through retrospective questions, for example leading individuals to report medical treatment that never happened [19, 20]. In this retrieval process, a telescoping effect may appear whereby the individual’s response for the survey reference period is combined with their experience from a previous period (backward telescoping) or with events after the reference period (forward telescoping) [2]. Fourth, especially in the case of traumatic events, respondents may give incorrect answers for fear of retribution from other community members or government authorities, but also due to trauma-induced recall errors related, for instance, to mental health coping mechanisms. The latter have been examined through the concepts of motivated forgetting [4, 11] and the dissociative amnesia model [6].

Recall error can pose major problems for analyses that rely on ex-post surveys to measure the consequences of conflict on health. With information about the same individuals for the periods post-conflict and before (or during) conflict, obtained through recall questions, a natural empirical strategy is to compare reported outcomes after the conflict with those reported for the previous period, contrasting average outcomes for individuals who were exposed and not exposed to certain conflict events, or for individuals who were exposed to different intensities of conflict violence. Since exposure to conflict cannot be randomised, studies have usually attempted to overcome challenges for inference by employing regression techniques for longitudinal data based on generalisations of difference-in-differences (DD) estimation [1]. This is a standard estimation methodology that, by comparing *changes* in outcomes for e.g. individuals exposed and not exposed to conflict violence, eliminates the confounding influence from any individual characteristics that can be considered time-invariant during the timeframe of analysis, such as genetics or risk attitudes [8].

Unfortunately, as discussed above, recall ability may change even in short periods also due to conflict exposure itself, potentially introducing biases for causal inference based on ex-post survey data, which cannot be addressed by DD or similar estimation approaches. People who were exposed to intense conflict violence may have had their recall ability affected differently (or misreport under different patterns, or refuse to respond or drop from the survey at different rates) than less exposed people, in a way that affects reported outcomes and the corresponding comparisons between these two groups. This makes it hard to support claims that differences in e.g. health outcomes have been driven by exposure to conflict. In the absence of carefully identified natural experiment settings that mimic randomisation of conflict exposure (which are relatively rare, but have been exploited in some research, cf. e.g. [1], recall bias jeopardises the robustness of the evidence produced by masking differences in outcome trends over time between “treatment” and “comparison” groups, unless patterns of recall ability and misreport changed similarly during the study period for all the groups under comparison [1, 7]. The degree of “randomness” of changes in recall patterns between compared individuals is, therefore, crucial for the reliability of conclusions drawn from studies using longitudinal health-related data from ex-post recall surveys.

In this paper, we argue that it is feasible to implement ex-post health surveys that collect recall data, for conflict-affected populations, which is reliable for empirical inference via standard regression methods, thus allowing robust conclusions to be drawn about the consequences of conflict exposure for health. We base our discussion on lessons from a large-scale longitudinal study that we conducted in Colombia about the consequences of the civil conflict for population health and the health system. We conducted two rounds of a household survey in the Colombian province of Meta in the years 2018 and 2019, after the peace agreement with the largest rebel group (FARC) was signed in 2016. Our survey collected information about health and socio-economic characteristics of the respondents and their households in the survey years, and also asked respondents to evaluate the same characteristics in the reference year of 2014 (i.e. before the peace agreement) through recall questions.

Our case study illustrates how strategies developed in the psychology and psychometric fields, in particular approaches based on “Flashbulb” test and test-retest strategies, can be used to ensure that recall questions in ex-post conflict surveys are appropriately framed to capture the phenomena of interest. Our study also illustrates how these test strategies can indicate which groups of health-related characteristics have been reliably captured (or not) via recall questions for the purposes of quantitative inference, when complemented by an assessment of differences in recall error patterns and attrition across surveyed groups. We draw key lessons for similar studies, such as the importance of having recall error testing strategies embedded from the outset into any ex-post health data collection instruments. Our main goal is for the lessons drawn from our study to contribute to the applied conflict and health researcher’s toolkit. As such, our paper makes a much-needed contribution to the still incipient knowledge base about strategies to measure and improve the validity of health surveillance systems, survey and data collection methods in conflict and emergency settings [22].

The remainder of this paper is organised as follows. The “Methods” section outlines the problems imposed by recall bias for reliable inference about the health effects of conflict using survey data, and proposes strategies to test for and address recall bias. The section “Health survey data collection and recall bias in a (post-)conflict setting: a case study of the Colombian conflict and peace process” summarises the context of our case study in Colombia; the large-scale longitudinal survey (CONPAS) conducted by our research team; and describes how the recall bias tests suggested and other bias mitigation strategies were applied in the CONPAS case. The “Results” section presents the results of the application of these strategies to address recall bias in our CONPAS data. The “Discussion and conclusions” section discusses these results and concludes with key lessons for related research.

## Methods

We begin this section with a more detailed presentation of the problem imposed by recall bias for applied survey-based research, focusing on the case of conflict and health research. This is followed by the presentation of the two complementary approaches that we propose to identify and address recall bias—the Flashbulb test and test-retest strategies—for the general case of conflict surveys. This provides the basis for discussing the adaptation of these strategies to our own health survey in Colombia.

### The problem of recall bias in empirical analyses of conflict effects

Consider the following empirical specification:

1 $$y_{i} = \alpha_{0} + \alpha_{1} D_{i} + \mu_{i}$$

where $y_{i}$ is the health outcome for individual *i*, $\alpha_{0}$ is the intercept, $D_{i} \in \left\{ {0,1} \right\}$ is an indicator that takes the value of 1 for a geographical area where conflict was present and zero for any other non-affected geographical area (i.e. all individuals living in an area with conflict are assumed to be “exposed” to conflict violence), and $\mu_{i}$ subsumes all types of errors. In this basic specification, $\alpha_{1}$ measures the effect of conflict on the health outcome and is the parameter of interest for the applied researcher.

If the presence of conflict was randomly assigned across geographical areas, as in an experiment, then $\mu_{i}$ would be random, identically and independently distributed $\mu_{i} \;\sim\;N\left( {0,1} \right)$. This would imply, in practice, that all observable and non-observable individual characteristics are also distributed randomly across conflict and non-conflict zones. In this case, a direct computation of difference in mean health outcomes would be enough to obtain an unbiased estimate of $\alpha_{1}$, in principle without the need of including control variables to capture observed individual characteristics, or of adopting non-experimental estimation models to account for unobserved characteristics.

However, the use of retrospective survey data about individual outcomes for estimating the model above adds another potential source of error, namely recall error, which can bias the estimation of $\alpha_{1}$ even in a randomised study scenario. This is because recall errors may be affected by memory functioning that, in turn, may be affected by conflict intensity. Let’s assume, more realistically, that there are two types of errors, as follows:

2 $$\mu_{i} = \omega_{i} + \varepsilon_{i}$$

where $\omega_{i}$ is the survey recall error, defined as inaccuracies in the retrieving process of past events that affect the survey responses by individuals, and $\varepsilon_{i}$ subsumes all other observed and unobserved errors, for now assumed to be random $\varepsilon_{i} \;\sim\;N\left( {0,1} \right)$.

In a model with no covariates, $y_{i} = \alpha_{0} D_{i} + \omega_{i} + \varepsilon_{i}$, if recall errors are not correlated with the presence of conflict (i.e. recall errors are randomly distributed across conflict and non-conflict areas), $cov\left( {D_{i} ,\omega_{i} } \right) = 0$ and thus recall errors will not bias $\alpha_{1}$. However, since exposure to conflict may well be correlated with individual characteristics (e.g. socioeconomic status), a more realistic modelling approach is likely to be one that accounts at least for a set $X_{i}$ of observed individual covariates:

3 $$y_{i} = \alpha_{0} + \alpha_{1} D_{i} + X_{i} ^{\prime}\alpha_{x} + \omega_{i} + \varepsilon_{i}$$

In this model with covariates, the condition required for recall errors not to bias $\alpha_{1}$ is that the conditional independence assumption, $cov\left( {D_{i} |X_{i} ,\omega_{i} } \right) = 0$, holds in the data. This means that recall errors are randomly distributed across conflict and non-conflict areas, once we control for the observable characteristics.

We propose below complementary approaches to assess the presence of non-random recall bias in studies of conflict effects that use retrospective longitudinal survey data, analysed through the standard model as in Eq. (3). Although, in most situations, the analyst would still need to deal empirically with the existence of potential bias due to *other* unobservable confounders influencing $\varepsilon_{i}$ (e.g. underlying baseline differences in genetic or other health determinants), understanding how recall error patterns vary in the study population would help the analyst in assessing the likelihood that the conditional independence assumption holds in the data. It would also help in considering empirical strategies to address recall error should it be present in a given setting.

### Flashbulb test of recall bias

We suggest here a recall bias test strategy that is based on the concept of flashbulb memories developed in the psychology literature. The original “Flashbulb Memory” concept hypothesises that major events, such as natural tragedies or traumatic events, should be recalled vividly and reliably, and that these events trigger in the subject not only memories of the main event in the long term, but also of other personal circumstances of the subject at the time of such event. Brown and Kulik [5], in their seminal paper, use as an example the US President John F. Kennedy’s assassination, arguing that “Almost everyone can remember, with an almost perceptual clarity, where he was when he heard, what he was doing at the time, who told him, what was the immediate aftermath, how he felt about it, and also one or more totally idiosyncratic and often trivial concomitants” (p. 73). Further work by Wright et al. [27] analysed two major events in the UK, namely Margaret Thatcher’s resignation and the Hillsborough football disaster, in the context of a population survey, finding that in the longer term (three to four years after the events) even these memories can somehow fade, with only a small percentage of the population being able to remember these events vividly.

For this study, our interest does not lie in how accurately survey respondents can recall specific events per se, but rather in whether the patterns of inaccuracies in retrieving such events, or the forgetting process, differ between survey respondents living in areas more and less affected by conflict violence. We build on the Flashbulb Memory concept to construct a simple but novel recall bias test, which we call Flashbulb recall test, to assess how forgetting and memory functioning can affect inference from conflict and health survey responses. More specifically, we propose the use of a well-known past event, that can be corroborated objectively, to test if recall errors differ across areas affected and not affected by conflict, between two time periods generally defined here as pre- and post-peace.

The presence of recall error can be expressed empirically as:

4 $$\omega_{i} = \left\{ {\begin{array}{*{20}c} {0 \,if\, Z_{t - 1} = \hat{Z}_{t - 1,it} } \\ {1\, if \,Z_{t - 1} \ne \hat{Z}_{t - 1,it} } \\ \end{array} } \right\}$$

where $Z_{t - 1}$ is a well-known past event occurred at time “t − 1” (pre-peace) and $\hat{Z}_{t - 1,it}$ denotes the recall of the event by individual *i* at the time of the post-peace survey “t”. We can then test the conditional independence assumption in a model with covariates, $cov\left( {D_{i} |X_{i} ,\omega_{i} } \right) = 0$, by estimating the following regression:

5 $$\omega_{i} = \beta_{0} + \beta_{1} D_{i} + X_{i} ^{\prime}\beta_{x} + \varphi_{i}$$

where $\omega_{i} \in \left\{ {0,1} \right\}$ is an indicator that takes the value of zero if individual *i* retrieves accurately the past event, and 1 if the individual retrieves the event inaccurately (see Eq. 4),$\beta_{0}$ is the intercept, $D_{i} \in \left\{ {0,1} \right\}$ is an indicator that takes the value of 1 for conflict zones (zero otherwise),$X_{i}$ contains the control variables, and $\varphi_{i}$ is the error term.

Coefficient $\beta_{1}$ is the parameter of interest for this test of the conditional independence assumption: if we cannot reject the null hypothesis that $\beta_{1}$ is equal to zero (i.e. if $\beta_{1}$ is not statistically significant at conventional levels), then there is evidence to support that recall errors are distributed randomly across conflict and non-conflict areas, thus mitigating concerns about recall bias affecting the parameter $\alpha_{1}$ in Eq. (3). Conversely, rejecting the null hypothesis that $\beta_{1}$ is equal to zero would point to the presence of recall errors that are non-randomly distributed between conflict and non-conflict zones, and thus to potential recall bias.

This test implicitly assesses the patterns of memory functioning across surveyed populations in conflict and non-conflict zones. We expect that, as time passes, the normal forgetting of events will affect the retrieving process for all survey respondents, but if forgetting was different in conflict zones (say “higher” due to e.g. motivated forgetting, dissociative amnesia or even physical brain damage), then we would expect to find a statistically significant estimate of $\beta_{1}$, indicating potential recall bias for the estimation of conflict effects on health outcomes.

### Test-retest assessment of recall bias

Even if, on average, patterns of recall ability do not seem to differ across populations exposed and not exposed to conflict violence, it is still possible for recall errors to be systematically different across these populations at least for specific modules of a health survey. This could be the case if individuals exposed to conflict violence have a different attitude to responses about certain topics, which mediates their recall process.

To test for this possibility, we suggest an adapted test-retest method that is implemented by comparing the individual responses about a single past event that occurred in the pre-peace period “t − 1”, where those responses are provided by the person in two different post-peace survey rounds, “t” and “t + 1”. The presence of recall error for a specific survey question (or set of questions), specifically defined as discrepancy between the two responses provided by the same individual, can then be expressed as:

6 $$\omega_{i} = \left\{ {\begin{array}{*{20}c} {0\, if\, \hat{Z}_{t - 1,it} = \hat{Z}_{t - 1,it + 1} } \\ {1\, if \,\hat{Z}_{t - 1,it} \ne \hat{Z}_{t - 1,it + 1} } \\ \end{array} } \right\}$$

where $\hat{Z}_{t - 1,it}$ is the recall of the past event (which took place in the pre-peace period “t − 1”) collected in the first post-peace survey in “t”, while $\hat{Z}_{t - 1,it + 1}$ is the recall of the same event collected in the second post-peace survey in “t + 1”. We can then test the conditional independence assumption in a model with covariates, $cov\left( {D_{i} |X_{i} ,\omega_{i} } \right) = 0$, through the following specification:

7 $$\omega_{i} = \gamma_{0} + \gamma_{1} D_{i} + X_{i} ^{\prime}\gamma_{x} + \varphi_{i}$$

By contrast with Eq. (5), $\omega_{i}$ now refers to the coincidence between responses, gathered from two post-peace survey rounds, about an event that happened in the pre-peace period specifically to the individual (e.g. an illness episode), rather than using as a reference point a general past event that can be verified objectively. This test-retest approach thus involves a higher degree of subjectivity, since the analyst does not know what the “correct” response is for a given individual. Nevertheless, in order to assess the likelihood of recall bias in the estimation of conflict effects through parameter $\alpha_{1}$ in Eq. (3), it suffices to test the statistical significance of coefficient $\gamma_{1}$ in Eq. (7). Non-rejection of the null hypothesis that $\gamma_{1} = 0$ would indicate that even if recall errors are present for a set of questions in the survey, these errors are likely to be distributed randomly across conflict and non-conflict areas, hence increasing the analyst’s confidence that the conditional independence assumption holds and that recall bias is less likely to be a factor. Rejection of the null hypothesis would, conversely, offer support to the possibility of recall bias influencing comparisons of survey responses between individuals in conflict and non-conflict zones.

A useful feature of this test-retest approach is the possibility of its separate application to different modules of a health survey, allowing the analyst to identify specific sets of questions likely affected by recall bias. This can help the researcher in selecting empirical methods that may be more appropriate than DD estimation for analysing specific topics, as well as in interpreting the reliability of specific empirical conclusions. However, this test-retest approach is data-hungry, in that it requires individual responses to the same questions to be collected in at least two different periods.

### Extension to the case of populations affected by multiple degrees of conflict intensity

The exposition so far has focused on comparisons of outcomes for individuals living in areas affected versus not affected by conflict violence. We show here that it is trivial to extend the suggested recall bias tests to the case of comparisons across individuals living in areas affected by different *degrees* of conflict intensity instead.

Consider the case of *k*=1,…,*K* groups of geographic areas, where areas within each group were affected by the same degree of conflict intensity, with groups ranging from unaffected to severely affected areas. A slightly modified estimating equation for both recall tests, for the case of no covariates, is:

8 $$\omega_{i} = \delta_{0} + \delta_{1} D_{1i} + \delta_{2} D_{2i} + \cdots + \delta_{k - 1} D_{k - 1i} + \varphi_{i}$$

where $D_{1i} ,D_{2i} , \ldots ,D_{k - 1i}$ denote a set of categorical variables representing each degree of conflict intensity compared to the reference category $D_{ki}$. The probability of recall error in the reference category is captured by the intercept term, i.e. ${\text{Pr}}(\omega |D_{k} ) = \hat{\delta }_{0}$, where $\hat{\delta }_{0}$ is the ordinary least-squares estimate of $\delta_{0}$. The probability of recall errors in the other conflict categories is given by their corresponding estimated coefficients plus the probability of the reference category, i.e. $\Pr \left( {\omega {|}D_{1} } \right) = \hat{\delta }_{1} + \hat{\delta }_{0} ,{\text{ Pr}}\left( {\omega {|}D_{2} } \right) = \hat{\delta }_{2} + \hat{\delta }_{0} , \ldots ,{\text{ Pr}}\left( {\omega {|}D_{k - 1} } \right) = \hat{\delta }_{k - 1} + \hat{\delta }_{0}$. A straightforward test for whether recall errors are statistically the same across conflict intensity categories is then to test the null hypothesis of $\hat{\delta }_{1} = \hat{\delta }_{2} = \ldots = \hat{\delta }_{k - 1} = 0$.

The inclusion of observed covariates in the estimating equation does not materially affect the conclusions above. Consider:

9 $$\omega_{i} = \delta_{0} + \delta_{1} D_{1i} + \delta_{2} D_{2i} + \cdots + \delta_{k - 1} D_{k - 1i} + X_{i}^{\prime } \delta_{x} + \varphi_{i}$$

Although $\delta_{0}$ in this specification no longer reflects the probability of recall error in the reference category, the estimated coefficients of each categorical variable still represent the marginal effects of recall errors for each conflict intensity category compared with the reference category $D_{ki}$, conditional on covariates. As before, we can test whether the distribution of recall errors is the same for populations in areas affected by different degrees of conflict intensity, by testing statistically the null hypothesis of $\hat{\delta }_{1} = \hat{\delta }_{2} = \ldots = \hat{\delta }_{k - 1} = 0$. Note that in the event of rejection of this null hypothesis, the researcher can conduct pair-wise testing to identify pairs of geographical areas for which the conditional independence assumption seems to hold—and, therefore, any population sub-groups for which comparisons of outcomes through DD estimation approaches may be more reliable.

## Health survey data collection and recall bias in a (post-)conflict setting: a case study of the Colombian conflict and peace process

### Background: the Colombian conflict and peace process

Since 1948 an estimated 220,000 people have died in Colombia and around 8 million have been displaced due to one of the longest civil conflicts in the world, with an estimated 7 million direct victims of conflict violence between 1985 and 2015 [13, 26]. Guerrilla groups, mainly the Revolutionary Armed Forces of Colombia (FARC) who were active in the majority of the country’s provinces, along with a number of other actors including paramilitary groups, have been involved in cross-fire, kidnappings, massacres, torture, extortion and other forms of violence and human rights violations. The conflict has led to a large burden of chronic conditions and mental health illnesses, poor healthcare infrastructure in conflict-affected areas, and worsened health inequalities due to the conflict’s heavier burden on poor and rural citizens [21].

Following an extensive negotiation process, a peace accord between the FARC and the Colombian government was agreed in November 2016. Despite ongoing reports that other armed groups remain active in certain areas of the country, where violence levels are still high, former FARC guerrillas have largely adhered to the demobilisation process outlined in the peace accord, leading to a reduction in violence levels after the peace agreement particularly in the areas where FARC were historically active [9].

### The *Conflict, Peace and Health* (CONPAS) survey

#### Study site

We planned primary survey data collection about the health and wellbeing of Colombians before and after the peace accord, as part of a large study about the health-related consequences of the Colombian conflict.^1^ We selected the central province of Meta for our fieldwork mainly because historically it is one of the areas that saw the worst of FARC-related conflict violence, and that therefore stood to gain substantially from FARC demobilisation after the accord. Moreover, despite it having been affected by the armed conflict from an early stage, the degrees of persistence and intensity of conflict violence have varied considerably across Meta’s 29 municipalities. According to the classification developed by the Colombian Conflict Analysis Resource Center (CERAC), four municipalities can be categorised has having been “heavily affected” by high intensity conflict violence; 22 municipalities have been “lightly affected” (judged by intermittent periods of violence, with variations of high and moderate conflict intensity among municipalities within this category); and three municipalities can be considered “not affected” directly [10]. For our study, we considered the province’s capital city Villavicencio (which contains more than half of the province’s population) as a further separate category of conflict intensity. This is because, despite it belonging to the “heavily affected” CERAC category, its categorisation is mostly due to Villavicencio’s role in receiving large contingents of people from other municipalities, who were displaced by conflict violence and were in search of better economic opportunities in the capital. As such, Villavicencio’s conflict situation differed markedly from that of the other three “heavily affected” municipalities, where armed violence episodes were a common feature before the 2016 peace accord.^2^

#### Data collection process, participants and questionnaire

The first round of the CONPAS—Conflict, Peace and Health (*Conflicto, Paz y Salud*)—household survey was conducted in all 29 municipalities of Meta in late 2018. The survey questionnaire and consent forms were approved by the relevant ethics boards of Universidad de los Andes (Colombia) and the University of York (UK). Informed consent was taken from each household prior to the questionnaire being administered. Data collection was directed to the household heads or (in their absence) a resident aged 18 years or older. For questions about child health, the mother or another adult female was asked to provide responses. Information was collected electronically by a local survey company and monitored daily by field supervisors for any inconsistencies. Sampling design ensured that CONPAS information is representative of the total, urban and rural populations of Meta, as well as of the populations living in municipalities “heavily”, “lightly” or “not affected” by conflict violence and in the capital Villavicencio.

The CONPAS questionnaire explores various aspects of people's health and wellbeing, such as forced displacement, sociodemographic and economic characteristics, general health status, health-related quality of life, mental health, disability, health expenditures and access to health services, among others (see Additional file 1: Appendix Table S1 for a summary description of CONPAS modules). To enable analyses of health changes in these domains before and after the FARC peace accord, the first CONPAS round collected contemporaneous information (i.e. referring to year 2018) as well as recall information for the same domains referring to year 2014 (i.e. pre-peace accord). The resulting CONPAS round 1 dataset includes information for 1309 households, with 4410 household members, among which 350 children between 3 and 5 years old. The initial sample of 1309 households included a 10% oversample to account for possible attrition (loss-to-follow-up) in the second round, while still maintaining representativeness of the population at the levels of the Meta province, urban/rural populations and groups of municipalities by level of conflict.

The second round of the CONPAS survey was conducted in late 2019. The enumerators set out to visit the same 1309 households interviewed for the first round. However, data collection was more challenging than in 2018, in particular due to higher rates of refusal among respondents to participate again in the survey.^3^ Subsequent attempts were made by the survey implementing company over the phone to contact and schedule visits to these households, and to track and interview respondents who moved within Meta since the first round, resulting in some sample recovery. In total, we obtained 1106 complete interviews in the second round, representing 84.6% of the original sample of respondents. The CONPAS round 2 questionnaire contained the same questions as in the first round, but asked the main respondent questions referring only to the year 2019, by contrast with the first round where recall questions referring to year 2014 were also asked. Additional file 1: Appendix Table S2 presents descriptive statistics for the CONPAS respondents, by survey year.

#### Recall questions about health and living standards in 2014: mitigating bias ex-ante

We adopted complementary strategies to aid recall for survey questions referring to year 2014. Firstly, where household composition permitted, the answers to these questions provided by the main respondent were confirmed with a second adult respondent in the household. This was particularly useful for questions related to living standards (e.g. dwelling conditions, expenditures, income, employment), household composition, displacement history and some health-related information (e.g. healthcare utilisation and health status of the main respondent and children). Where there was disagreement between the initial responses provided by the main and second respondents (which ended up occurring in a very small number of cases, less than 1% of responses), enumerators encouraged individuals to reach a consensus response.

Secondly, enumerators were trained to ask the participant, before conducting the 2014 questions, to recall a personal or family event that occurred in that year. This was followed by prompts to help participants situate their responses in time, before the start of each relevant survey module. These prompts contained mentions to two noteworthy events that occurred in 2014 and that were easily discernible for respondents: the presidential election (won by Juan Manuel Santos, who eventually led the 2016 peace accord process) and the FIFA 2014 football World Cup (where Colombia’s national team reached the quarterfinals for the first time in its history).^4^ The phrasing of each contemporaneous question was then slightly modified to capture the corresponding 2014 information (see Additional file 1: Appendix Table S3 for example questions). Our procedure followed evidence that recall can be aided by using event timelines, based on well-known national events or individual/local history, to stimulate the respondent’s memory and accurately situate personal events in time [7].

To mitigate recall errors for the “contemporaneous” questions referring to years 2018 and 2019, relatively short recall times were preferred, most of which are usual practice in household health surveys, e.g. the Demographic and Health Surveys (including its Colombian version).^5^

#### Attrition patterns: is attrition between CONPAS survey rounds associated with conflict exposure?

CONPAS presented high acceptability with usually low item non-response rates (< 10%). However, we mentioned previously that the second CONPAS round conducted in 2019 was affected by an attrition rate of about 15.4% (203 individuals), which is higher than the 10% oversampling included in the first round of CONPAS to mitigate loss-to-follow-up. If individuals with certain characteristics—e.g. those who lived in areas where conflict intensity was the highest pre-2016 accord—were more likely to drop out from the sample between survey rounds, this would introduce a source of bias for any comparisons of outcomes between people affected by different levels of conflict violence, by under-representing the recall responses of those most affected by the conflict. On the other hand, if attrition between rounds occurred in such a way that was not correlated with conflict exposure or key individual observable characteristics, such as education or socio-economic status, it is more likely that people who dropped out from the sample also had similar unobservable characteristics (on average) to those who remained in the sample. Random loss-to-follow-up would still allow standard regression-based panel data methods like DD to recover the “true” effects of conflict exposure on individual health, using recall data as that in our CONPAS survey.

In order to estimate the association between probability of sample attrition and exposure to conflict violence, we ran a simple ordinary least-squares linear regression of a binary loss-to-follow-up dependent variable (which assumed the value of one if the individual dropped out of the sample in 2019, zero otherwise) on a set of binary variables representing each of the levels of conflict intensity in the respondent’s municipality of residence (“heavily affected”, “lightly affected” and Villavicencio, with “not affected” as the reference category). We also included in this regression a set of individual demographic controls (displaced status, sex, age, education, employment and marital status), along with the municipality’s population density to prevent undue influence in the estimation results of a few Meta municipalities with large populations.

Additional file 1: Appendix Table S6 shows the estimation results for the association between probability of attrition in 2019 and conflict intensity level in the municipality of residence. Overall, the message from these analyses is that attrition between CONPAS rounds is not systematically related to the level of conflict intensity experienced by the respondent in their area of residence, once we control for demographic factors and population density. This result provides reassurance that the results of our CONPAS recall bias tests (described below) will not be driven by a selected sample of respondents in the 2019 survey round. It also highlights the importance of controlling for those basic demographic and population density characteristics in estimations of the health effects of conflict using CONPAS survey data.

#### Flashbulb test of recall bias for CONPAS

For our adapted Flashbulb test approach, we added to the 2019 CONPAS questionnaire a set of questions about a well-known past event, Colombia’s national football team performance in the 2014 FIFA World Cup (Table 1). The aim was to assess patterns of deviations from the correct responses, alongside self-reported levels of confidence in these recall responses and the degree of importance attributed by the respondent to Colombia’s participation in the 2014 football World Cup. The latter allows us to compare recall ability across individuals living in areas with different pre-accord conflict intensity, while accounting also for differences in the subjective relevance of the “flashbulb” event for these individuals.

**Table 1:** Flashbulb recall test questionnaire—CONPAS 2019

Question ID	Question phrasing	Response options
701	How clearly do you recall Colombia's participation in the 2014 FIFA Football World Cup in Brazil?	0. Does not recall it 1. Recalls it vaguely 2. Recalls it more or less clearly 3. Recalls it very clearly 4. Recalls it vividly
702	At that time, how much importance did you attach to the event of Colombia's participation in the 2014 FIFA Football World Cup in Brazil?	0. Did not consider it to be of any importance 1. Did not consider it to be very important 2. Considered it somewhat important 3. Considered it very important 4. Considered it extremely important
703	What stage did Colombia reach at the 2014 FIFA Football World Cup in Brazil?	1. Final 2. Semi-final 3. Quarterfinals 4. Round of 16 5. Did not get through the group stage 6. Does not know
704	On a scale of 1 to 5 (where 1 is not at all confident and 5 is extremely confident), how confident do you feel about this recollection?	1 2 3 4 5

We implemented our Flashbulb test by running ordinary-least squares linear regressions where the dependent variable was a binary indicator taking the value of one for an incorrect response, zero otherwise, referring to the question “What stage did Colombia reach at the 2014 FIFA Football World Cup in Brazil?” (the correct answer is the quarterfinals). The main regressors of interest were the set of binary variables representing the levels of conflict intensity in the respondent’s municipality of residence (“not affected” was the reference category). We ran separate regression specifications as in Eq. (9), which included a basic set of individual demographic controls (characteristics that for adult respondents are less likely to be correlated with conflict intensity during the survey period, namely sex, age and education), the municipality’s population density, the self-reported level of confidence in recall responses about the World Cup event, and the self-reported degree of importance attributed to the World Cup event.

#### Test-retest assessment of recall bias for CONPAS

Although CONPAS did not ask about traumatic events directly, it could be the case that some people failed to report illnesses or healthcare episodes that did occur, if those episodes were vividly associated with exposure to conflict events. Such misreporting (including by forgetting) could therefore reflect a coping mechanism.

We implemented an adapted version of the test-retest approach to test for differences in recall error patterns across areas. We selected 17 questions that referred to year 2014 and which were included in the 2018 survey round, spanning all CONPAS modules, to be asked again from all respondents during the 2019 survey round.^6^ The questions selected in each CONPAS module for this test-retest approach are listed in Additional file 1: Appendix Table S7; these were selected to cover key health and wellbeing aspects being investigated in our larger study. The idea was to have clear indications both about how stable recall reporting referring to year 2014 is, and also whether patterns of recall error or instability for specific survey modules seem to be linked systematically to the degree of pre-accord conflict intensity in the respondent’s municipality of residence.

For each selected question referring to year 2014, we ran a separate ordinary least-squares linear regression where the dependent variable was either a binary variable taking the value of one if the participant’s 2018 and 2019 responses differed, zero otherwise (for yes/no questions), or the absolute numeric difference between 2018 and 2019 responses (for numeric questions). The key regressors of interest for this test were the set of binary variables representing the levels of conflict intensity in the respondent’s municipality of residence (“not affected” was the reference category), complemented by the basic individual demographic controls (sex, age and education) and the municipality’s population density, following the specification in Eq. (9).

## Results

### Flashbulb recall test: are recall ability patterns in CONPAS linked to conflict exposure?

Table 2 shows the results of our proposed Flashbulb test, applied to CONPAS data. In the first column we report the regression results with no control variables, as in Eq. (8), where we find that the estimated coefficient for heavily affected municipalities ($\hat{\delta }_{3} = 0.0997$) is statistically different from zero, suggesting that recall error patterns are systematically different for individuals living in highly affected areas compared to their counterparts living in unaffected areas.

**Table 2:** Flashbulb recall test regression results for CONPAS survey data

	(1)	(2)	(3)
*Conflict intensity level*			
Not affected	Ref.	Ref.	Ref.
Villavicencio	0.0194 (0.0512)	0.0687 (0.0505)	0.0568 (0.0462)
Lightly affected	0.1064 (0.0541)	0.0666 (0.0465)	0.0639 (0.0420)
Heavily affected	0.0997** (0.0370)	0.0608 (0.0371)	0.0549 (0.0351)
*Control variables*			
Demographics	No	Yes	Yes
Population density	No	Yes	Yes
Event importance	No	No	Yes

Demographics include sex, age and education. Event importance refers to the responses to the questions “How clearly do you recall Colombia's participation in the 2014 FIFA Football World Cup in Brazil?” and “At that time, how much importance did you attach to the event of Colombia's participation in the 2014 FIFA Football World Cup in Brazil?”, included as categorical variables for each response option. **p < 0.05, ***p < 0.01. Taylor-linearized standard errors in parentheses account for complex survey sample design

However, these differences in recall patterns disappear once the differences across areas in basic observable characteristics are accounted for, as in Eq. (9). Column 2 includes the set of demographic (sex, age and education) and population density controls, showing no estimated coefficients that are statistically different from zero (i.e. $\hat{\delta }_{1} = \hat{\delta }_{2} = \hat{\delta }_{3} = 0$). The model estimated in column 3 includes the previous covariates but controls also for the subjective confidence in recall ability and importance attributed by individuals to the FIFA World Cup event, with similar results as in column 2, thus supporting again the hypothesis that recall error patterns are not systematically related to the degree of conflict intensity to which individuals were exposed in their area of residence. This increases confidence in the analysis of CONPAS data through standard approaches such as DD (where controls for basic observable confounders are included) for causal inference about conflict effects among the study population.

### Test-retest assessment: are misreporting or recall stability patterns in CONPAS linked to conflict exposure?

Table 3 presents the results of our test-retest assessment of recall bias for 17 questions across the seven CONPAS modules.^7^ For example, the first coefficient in the first column, $\hat{\delta }_{1} = 0.0249$, is not statistically different from zero. This indicates that there are no systematic differences in misreporting or recall stability patterns regarding access to electricity in 2014, between respondents from Villavicencio and those from areas not affected by conflict, once basic observable differences across areas are controlled for. The same situation is observed for responses from lightly or heavily affected areas, compared to unaffected areas.

**Table 3:** Test-retest regression results for CONPAS survey data

	Household living standards					Household expenditures		Demographic and socioeconomic conditions	
	(1)	(2)	(3)	(4)	(5)	(6)	(7)	(8)	(9)
	Electric power	Gas	Water	Sewerage	Rubbish collection	Total	Health	Marital status	Education
*Conflict intensity level*									
Not affected	Ref.	Ref.	Ref.	Ref.	Ref.	Ref.	Ref.	Ref.	Ref.
Villavicencio	0.0249 (0.0284)	− 0.0355 (0.0518)	0.0173 (0.0486)	0.0466 (0.0496)	− 0.0225 (0.0448)	− 109,800** (41,526)	− 71,678*** (19,238)	0.0405 (0.0399)	− 0.0252 (0.0436)
Lightly affected	0.0513 (0.0306)	− 0.0296 (0.0472)	0.0450 (0.0424)	0.0226 (0.0399)	0.0111 (0.0412)	− 70,254 (41,619)	− 23,102 (19,872)	− 0.0454 (0.0297)	0.0322 (0.0371)
Heavily affected	0.0468 (0.0340)	− 0.1339*** (0.0442)	− 0.0442 (0.0415)	− 0.0538 (0.0378)	− 0.1004** (0.0390)	− 29,467 (37,365)	− 21,401 (17,162)	− 0.0256 (0.0339)	0.0176 (0.0377)
*Control variables*									
Demographics	Yes	Yes	Yes	Yes	Yes	Yes	Yes	Yes	Yes
Population density	Yes	Yes	Yes	Yes	Yes	Yes	Yes	Yes	Yes

	General health/health-related quality of life				Mental health			Disability		Alcohol and nicotine consumption		
	(10)	(11)	(12)	(13)	(14)	(15)	(16)	(17)	(18)	(19)	(20)	(21)
	General health	Hospitalization	Mobility impairment	General health (visual scale)	Headaches	Trouble sleeping	Interference in thinking	Difficulty standing	Difficulty walking	Alcohol consumption	Injury due to drinking	Smoking
*Conflict intensity level*												
Not affected	Ref.	Ref.	Ref.	Ref.	Ref.	Ref.	Ref.	Ref.	Ref.	Ref.	Ref.	Ref.
Villavicencio	− 0.0048 (0.0476)	− 0.1420*** (0.0383)	− 0.0373 (0.0410)	− 0.0643 (0.0408)	− 0.0684 (0.0348)	0.0335 (0.0322)	− 0.4407 (2.0408)	− 0.0462 (0.0471)	− 0.0923 (0.0450)	0.0055 (0.0594)	0.0131 (0.0191)	− 0.0538 (0.0307)
Lightly affected	− 0.0726 (0.0418)	− 0.0850** (0.0387)	− 0.0204 (0.0393)	− 0.0596 (0.0406)	− 0.0398 (0.0328)	0.0233 (0.0325)	0.5528 (1.6169)	− 0.0138 (0.0547)	− 0.0012 (0.0446)	− 0.0917 (0.0467)	− 0.0134 (0.0110)	− 0.0679** (0.0286)
Heavily affected	− 0.1238** (0.0451)	− 0.0456 (0.0422)	0.0368 (0.0406)	− 0.0298 (0.0342)	− 0.0223 (0.0327)	0.0509 (0.0301)	2.2384 (1.5275)	0.0090 (0.0399)	0.0433 (0.0412)	0.0606 (0.0553)	0.0401** (0.0173)	− 0.0841*** (0.0279)
*Control variables*												
Demographics	Yes	Yes	Yes	Yes	Yes	Yes	Yes	Yes	Yes	Yes	Yes	Yes
Population density	Yes	Yes	Yes	Yes	Yes	Yes	Yes	Yes	Yes	Yes	Yes	Yes

Demographics include sex, age and education. **p < 0.05, ***p < 0.01. Taylor-linearized standard errors in parentheses account for complex survey sample design

The regressions suggest that, in most cases, discrepancies between survey rounds in the individual responses for the same recall question are not linked systematically to violence exposure levels, judged by estimated coefficients that are insignificant at conventional statistical levels. This is the case for key socioeconomic/demographic factors (marital status, educational attainment), mental health and disability aspects, as well as for most indicators of household living standards.

Yet there are a few instances where we find evidence that patterns of misreporting were indeed correlated with living in areas heavily affected by the conflict. For example, the third estimated coefficient in column 20 ($\hat{\delta }_{3} = 0.0401$) implies that individuals from heavily affected areas had a 4% higher probability (compared to people in unaffected areas) of providing a different response to the yes/no question about injury due to drinking in 2014, between the 2018 and 2019 survey rounds. The results also suggest that patterns of misreporting of household expenditures, hospitalisations and smoking behaviour are different for people living in Villavicencio or lightly affected areas compared to people living in unaffected areas.

The results in Table 3 are very informative about the sub-populations for which comparisons of outcomes are more likely to be free from—or affected by—recall bias. For instance, in the case of the alcohol abuse outcome measured as injury due to drinking, standard estimations of conflict effects using CONPAS data are more likely to be reliable (with respect to potential recall bias) if these estimations focus on comparisons restricted to individuals from Villavicencio, lightly affected and unaffected areas, for whom recall error and misreporting patterns seem to be randomly distributed.

## Discussion and conclusions

In this paper, we discuss key challenges related to the collection and analysis of ex-post health survey data for conflict-affected populations. We focus primarily on the issue of potential recall bias when survey respondents are asked, in a post-conflict period, about life events, health and living conditions experienced before or during the conflict. We show how in that context recall bias threatens causal inference about the health effects of conflict exposure on individuals, when analyses are conducted using standard quantitative methods. We then propose two complementary strategies based on methods developed in the psychology and psychometric literatures—the Flashbulb and test-retest approaches—to identify and address recall bias in ex-post health survey data. The test-retest method and the Flashbulb recall tests are complementary in the sense that, whilst the latter seeks to assess *general* recall ability and biases, the former focuses on the reliability of responses to *specific* items in the questionnaire that will be used (often as outcome variables) for the investigation of conflict effects. Through the case study of two rounds of a large-scale health survey (CONPAS), which we implemented in Colombia in the post-peace agreement period, but that included recall questions referring to the conflict period, we demonstrate how adapted versions of the Flashbulb and test-retest strategies can be used to test objectively for the presence of recall bias in (post-)conflict survey responses. Our case study also shows how these test strategies can be incorporated into post-conflict health surveys already in their design phase, accompanied by other ex-ante mitigation strategies for recall bias (e.g. confirmation of recall responses with more than one respondent and use of well-known events to situate respondents in time to aid recall), to increase the reliability of longitudinal health data analysis.

Our study makes a novel contribution to the field of applied health research in humanitarian settings. Previous studies have highlighted the glaring knowledge gaps in the areas of survey design, data collection and surveillance systems for research and policymaking in these settings. Grais et al. [12], in their review of field surveys in the Democratic Republic of Congo, found great variation in methodological quality and concluded that mechanisms to ensure sound survey design—including the trialling of novel survey methods—are urgently needed in the humanitarian field more generally. Ratnayake et al. [22] conducted an overview of health research in humanitarian crises, where they identified a generalised absence of reports on the performance, validity and evaluation of health survey and data collection methods, concluding that further work on these issues is paramount. In many humanitarian or resource-constrained settings, information that is key for allocating large investments in the health system, such as mortality, morbidity and healthcare seeking histories in a population (and even basic demographic information such as child age), is obtained from large scale surveys based on recall data, with important recall biases often identified [7]. The same applies to the gathering of information about events leading to forced displacement and for the determination of an individual’s refugee status [12].^8^ There is a critical need for better methods to determine how reliable recall survey data is in these situations, and to improve the reliability of such data collection, in order to better guide investment decisions in a given context. Our study contributes to fill precisely all of the above knowledge gaps, by providing practical lessons for the implementation of data collection efforts in humanitarian contexts where recall information, collected from primary surveys, is required to allow assessments of changes in health and wellbeing. The latter is an all-too-common situation faced by researchers, owing to the usual limitations of the information available from administrative and other data sources for periods of humanitarian crisis [7, 22].

The case of our CONPAS survey illustrates how health survey design can incorporate objective tools to permit: (1) the assessment (and improvement) of the performance of pre-post recall modules against formal reliability criteria, and (2) data analysis that identifies the survey domains, and sub-populations, for which standard empirical analysis of the information gathered is likely to yield more reliable conclusions. For example, the application of the Flashbulb and test-retest strategies to the CONPAS survey data indicated that, for most survey modules, recall ability and misreporting patterns do not vary systematically with the levels of conflict intensity in the areas where respondents live, implying that standard regression methods can alleviate concerns about recall bias for analyses of the causal effects of the Colombian conflict using such pre-post data. However, there was indication that misreporting patterns for some questions (e.g. alcohol consumption, household expenditures) were systematically different between respondents living in areas more and less affected by conflict violence. For these groups of questions, recall bias is more likely to lead to unreliable comparisons of conflict effects through standard empirical approaches such as difference-in-differences estimation. This result emphasises the importance of careful selection, by the applied health researcher, of appropriate comparison groups in standard longitudinal analyses. These comparison groups may need to be different depending on the domains (responses) being evaluated. The recall bias test strategies illustrated in our paper constitute a mechanism for selecting such comparison groups in a way that is transparent and informed by data evidence. Applied researchers can use the evidence from these tests either to obtain reassurance about the low importance of recall bias in their impact evaluation context, or to help interpret the results of their impact evaluations as lower or upper bound effects, after taking into account the identified direction and magnitude of existing recall biases.

Despite the challenges for gathering reliable recall information about conflict or emergency periods through surveys conducted after these events, our study shows that this can be done so as to permit highly informative analyses. We share useful guidance on this based on the lessons from our experience with CONPAS in Colombia. By doing so, our study offers related research a blueprint for survey design and data analysis, not only in the health field but also for development research more generally. A key lesson is the importance of embedding appropriate strategies to test and address recall bias into any relevant data collection tools, already during the survey design phase. This is particularly useful in settings where primary recall data collection is deemed necessary, but the researcher has reasons to believe that the phenomenon under study—e.g. exposure to conflict violence or another humanitarian disaster—may have influenced recall ability (memory functioning) more for certain groups of respondents, or even where contextual incentives may have been introduced for certain respondents to misreport past experiences (e.g. fear of reprisals within the community in post-conflict zones, or settings where specific individuals may believe that their responses will affect entitlement to social services or benefits [7].

There are, of course, caveats that must be considered for the application of our recall bias test strategies in other research contexts. First, the selection of any one question for use in the Flashbulb recall test should be done in a manner that is informed and appropriate to the particular research setting, but at the end such selection is unavoidably subjective.^9^ In principle, using a few different “flashbulb events” could be helpful to provide a more rounded assessment of recall bias through our Flashbulb recall test. But although questions about further “flashbulb events” could always be added to the survey questionnaire by researchers implementing Flashbulb tests, there needs to be a sense *a priori* that the events chosen are indeed important or noteworthy for (at least most of) the population of interest, so as to effectively assist with accurate memory retrieval of past personal events. Moreover, there is a limit to how many such questions can be added without becoming a burden to respondents, and encouraging inaccurate answers or non-responses. These concerns, and our knowledge of the Colombian context, led us to our choice of using only the FIFA World Cup as the basis for implementing our Flashbulb test. At this point, we must reiterate the importance of there being a strategy in place to mitigate recall error in any survey-based study (beyond just testing for its presence), and such strategy must be multidimensional. In our study, this strategy includes the use of other potential “flashbulb events” to situate respondents in time for the main questionnaire, namely: (a) asking the participant, before conducting the 2014 questions, to recall a personal or family event that occurred in that year; along with (b) also referring the respondent to the 2014 presidential election. Similar strategies should be adopted by researchers seeking to apply the recall test methods illustrated here to other contexts.

A second caveat for applying the learnings from our case study to other contexts is that incorporating the full suite of proposed ex-ante mitigation and ex-post test strategies for recall error hinges on the possibility of conducting more than one survey round with the same respondents. This is the case for the test-retest approach more specifically. The researcher also faces a trade-off when adopting the test-retest strategy, between the scope of survey modules scrutinised (the more questions and modules, the more informative the evidence gathered will be) and its cost (the more questions included in the test, the higher the cost in terms of respondents and interviewers’ time will be). It is therefore advisable for the researcher to focus primarily on certain survey modules that may be representative of the survey as a whole, and/or on modules containing questions considered of essential importance for the analysis planned. Although, for any given study, the most appropriate questions to be selected for test-retest will thus depend on the study’s main goals, the principles and application of the test-retest approach illustrated here are generalisable to a wide range of studies based on retrospective survey data. Furthermore, strategies such as the use of well-known events to situate respondents in previous periods of time and the Flashbulb test approach can be applied using a single survey round, representing readily available tools for the design of appropriate surveys and robust analysis of any retrospective health-related information collected.

## Supplementary Information

**Additional file 1.** **Table S1:** CONPAS questionnaire modules. **Table S2:** Selected demographic and socio-economic characteristics of CONPAS respondents, by survey year (2018 and 2019). **Table S3:** Sample of questions – CONPAS 2014 and 2018. **Table S4:** Cronbach’s alpha test results by CONPAS modules and survey years. **Table S5:** IRT reliability test results by CONPAS modules and survey years. **Table S6:** Probability of attrition in the second round of CONPAS. **Table S7:** Questions selected for the test-retest approach – CONPAS 2019.

## Data Availability

The dataset analysed during the current study is available from the corresponding author on reasonable request.

## Footnotes

1 “War and Peace: the Health and Health System Consequences of Conflict in Colombia”, 2018–2021, funded by the UK Joint Health Systems Research Initiative (MRC/ESRC/DFID/Wellcome Trust, Grant MR/R013667/1).

2 A final reason for choosing Meta was that, after the signing of the peace accord, the safety and security situation in its municipalities improved considerably for conducting fieldwork, favouring also interviewee follow-up.

3 Refusals were mainly due to issues such as increasing levels of urban violence and insecurity in Meta, and in some cases a perception of insufficient personal benefit from taking part in the study.

4 The general phrasing of these prompts before the start of each module was (translated from Spanish): *“Now I will ask you questions referring to two different time periods: the first one being the current period, and the second one being during year 2014, when the FIFA 2014 football World Cup was held in Brazil, and the presidential election took place, electing president Juan Manuel Santos”*.

5 We note that the CONPAS survey modules presented good internal consistency reliability, as assessed by standard psychometric measures, indicating that the items (questions) contained within each module complement each other well in their measurement of different aspects of the same health or wellbeing dimension of interest. Details are provided in the Additional file 1: Appendix.

6 We opted for a selection of questions, instead of asking all the 2014 recall questions again in the 2019 survey round, to reduce survey time in the second round, mitigate participant’s fatigue, and avoid withdrawals from the study based on participant’s refusal to respond all the numerous retrospective questions once again.

7 Table 3 shows results for 21 regressions (columns) because question one, about household living conditions, has five independent sub-questions.

8 We thank an anonymous referee for raising these points.

9 We thank an anonymous referee for raising this point.
