# Supplementary material for: Addressing recall bias in (post-)conflict data collection and analysis: lessons from a large-scale health survey in Colombia
Source: Confl Health. 2022 Apr 8;16:14. doi: 10.1186/s13031-022-00446-0 (PMC8994310; doi:10.1186/s13031-022-00446-0)
Supplement: Supplementary file 1 — Additional file 1. Table S1: CONPAS questionnaire modules. Table S2: Selected demographic and socio-economic characteristics of CONPAS respondents, by survey year (2018 and 2019). Table S3: Sample of questions – CONPAS 2014 and 2018. Table S4: Cronbach’s alpha test results by CONPAS modules and survey years. Table S5: IRT reliability test results by CONPAS modules and survey years. Table S6: Probability of attrition in the second round of CONPAS. Table S7: Questions selected for the test-retest approach – CONPAS 2019. [file 13031_2022_446_MOESM1_ESM.docx]

Additional file 1

Appendix (supplementary information)

**Table S1: CONPAS questionnaire modules**

| **Module** | **Name** | **Contents of questions** |
| --- | --- | --- |
| A | Household and individual identification | Identification data for the household and personal information of survey respondent. |
| B | Outcome of survey visit, type of supervision, and visit control | Date and hour of survey application and enumerator’s information. |
| I.A | Information about household members | Name, age, sex and date of birth of each household member; number of children and number of household members. |
| I.B | Control information: interviewed adults | Selected adult(s) to be interviewed by enumerator in the household. |
| II | Housing and living standards | Access to public services, sanitation, water and energy sources; materials of walls and floors; ownership of home appliances and other assets. |
| III | Household expenditures | Household expenditures in the past month, disaggregated into total, food and health expenditures; financial sources used to fund these expenditures. |
| IV.A | Internal displacement due to conflict  (Adults aged 18+) | Time period in the current address, household and area; changes in residence and household membership due to the conflict; current internal displacement due to the conflict. |
| IV.B | Demographic and socioeconomic conditions  (Adults aged 18+) | Ethnicity; highest education level achieved; current occupation; marital status; labour income; health insurance affiliation; participation in nutrition and health social programmes; perceived economic status of the household. |
| IV.C | General health  (Adults aged 18+) | Self-assessed health status; recent illness and hospitalisation; healthcare seeking behavior; type of facility and perceived quality for services used; reasons for not seeking or receiving health services. |
| IV.D | Mental health  (Adults aged 18+) | WHO Self-Report Questionnaire (SRQ-25) about psychosomatic symptoms and symptoms related to psychiatric disorders. |
| IV.E | Health-related quality of life  (Adults aged 18+) | EQ-5D-3L instrument assessing self-assessed health status, mobility capability, pain and mental distress (anxiety or depression). |
| IV.F | Disability  (Adults aged 18+) | World Health Organization Disability Assessment Schedule (WHODAS) instrument. |
| IV.G | Alcohol use disorder  (Adults aged 18+) | WHO Alcohol Use Disorders Identification Test (AUDIT) instrument. |
| IV.H | Nicotine dependence  (Adults aged 18+) | Fagerstrom Test for Nicotine Dependence instrument. |
| V.A | Demographic and socioeconomic conditions  (Children under 5 y.o.) | For each child (as reported by the main respondent): health insurance affiliation; participation in nutrition and health social programmes. |
| V.B | General health  (Children under 5 y.o.) | For each child (as reported by the main respondent): health status; recent illness and hospitalisation; healthcare seeking behavior; type of facility and perceived quality for services used; reasons for not seeking or receiving health services. |
| VI | Further contact details | Contact details of two additional people, outside of the household, who could be contacted to allow tracking the main respondent for future surveys, in case of change of address. |

**Table S2: Selected demographic and socio-economic characteristics of CONPAS respondents, by survey year (2018 and 2019)**

|  | 2018 (n=1309) | | 2019 (n=1106) | | |  |
| --- | --- | --- | --- | --- | --- | --- |
| Age, mean (SE) | 45.3 | (1.06) | 47.2 | (0.89) | |  |
| Male, n (%) | 600 | (39.6) | 508 | (39.1) | |  |
| Ethnicity, n (%) |  |  |  |  | |  |
| White | 652 | (43.8) | 473 | (42.7) | |  |
| Black/Mestizo | 217 | (14.8) | 192 | (15.0) | |  |
| Others | 530 | (41.4) | 441 | (42.3) | |  |
| Marital status, n (%) |  |  |  |  | |  |
| Married/co-habiting | 817 | (62.3) | 735 | (66.2) | |  |
| Single | 99 | (11.1) | 83 | (10.5) | |  |
| Separated/divorced/widowed | 393 | (26.6) | 288 | (23.3) | |  |
| Education, n (%) |  |  |  |  | |  |
| No education | 79 | (4.7) | 274 | (14.6) | |  |
| Primary | 535 | (29.9) | 396 | (35.3) | |  |
| Secondary | 439 | (39.6) | 259 | (29.4) | |  |
| Technical or higher | 256 | (25.7) | 177 | (20.7) | |  |
| Occupation, n (%) |  |  |  |  | |  |
| Employed | 462 | (28.0) | 414 | (30.8) | |  |
| Self-employed | 314 | (27.0) | 258 | (28.1) | |  |
| Unemployed/others | 533 | (45.0) | 434 | (41.1) | |  |
| Health expenditure, monthly mean (SE) | 80910 | (9059.3) | 62719 | | (6469.2) | |
| Area of residence, n (%) |  |  |  |  | |  |
| Municipal capital | 782 | (76.4) | 633 | (71.0) | |  |
| Urban town | 179 | (10.5) | 174 | (13.6) | |  |
| Rural town | 348 | (13.1) | 299 | (15.4) | |  |
| Conflict intensity level, n (%) |  |  |  |  | |  |
| Not affected | 294 | (2.0) | 257 | (2.3) | |  |
| Villavicencio | 300 | (51.0) | 228 | (47.4) | |  |
| Lightly affected | 409 | (41.9) | 342 | (44.4) | |  |
| Heavily affected | 306 | (5.1) | 279 | (6.0) | |  |

Note: Means, percentages (%) and standard error (SE) estimates account for complex survey sample design. Health expenditure in Colombian pesos.

**Table S3: Sample of questions – CONPAS 2014 and 2018**

**Panel A: Demographic and Socioeconomic Conditions module (IV.B) – selected questions**

| 🖐 Enumerator: *Read to the respondent: “Now I will ask you questions referring to two different time periods: the first one being the current period, and the second one being during year 2014, when the FIFA 2014 football World Cup was held in Brazil, and the presidential election took place, electing president Juan Manuel Santos.” If the respondent is not located in the period of time (2014), refer to a positive or negative event that could have occurred to the respondent such as: a wedding, birth of a loved one, death of a loved one, etc.”.* | | | | | 1.Current | 2.During 2014 |
| --- | --- | --- | --- | --- | --- | --- |
| **408** | | What is your marital status? | Married | | 1 | 1 |
|  |  |  | Cohabiting | | 2 | 2 |
|  |  |  | Separated/ divorced | | 3 | 3 |
|  |  |  | Widowed | | 4 | 4 |
|  |  |  | Single | | 5 | 5 |
| **409** | | What is the highest formal education level you attained? | None | | 1 | 1 |
|  |  |  | Preschool / Primary | | 2 | 2 |
|  |  |  | Secondary | | 3 | 3 |
|  |  |  | Technical /higher /postgraduate education | | 4 | 4 |
| **411** | Last month, how much did you earn from your work activities (including tips, commissions and bonuses, per diem, overtime and payments in kind)? | | Less than 1 minimum wage (MV) | | 1 | 1 |
|  |  |  | Between 1 and less than 2 MV | | 2 | 2 |
|  |  |  | Between 2 and less than 5 MV | | 3 | 3 |
|  |  |  | 5 or more MV | | 4 | 4 |
| **…** |  | |  | |  |  |
| **417ª** | 🖐 Enumerator: Could the information in chapter IVB be confirmed with a second respondent? | | | Yes 1 | No 2 | NA 3 |

**Panel B: General Health module (IV.C) – selected questions**

| 🖐*Enumerator: Read to the respondent: “Now I will ask you questions referring to two different time periods: the first one being the current period, and the second one being during year 2014, when the FIFA 2014 football World Cup was held in Brazil, and the presidential election took place, electing president Juan Manuel Santos.” If the respondent is not located in the period of time (2014), refer to a positive or negative event that could have occurred to the respondent such as: a wedding, birth of a loved one, death of a loved one, etc.”.* | | | | 1.Current | 2.During 2014 |
| --- | --- | --- | --- | --- | --- |
| **418** | How do you rate your health in general? | Excellent | | 1 | 1 |
|  |  | Very good | | 2 | 2 |
|  |  | Good | | 3 | 3 |
|  |  | Fair | | 4 | 4 |
|  |  | Poor | | 5 | 5 |
| **419** | During the last 12 months / During 2014, were you hospitalized for any illness or other health problem (except pregnancy, but including violence events)?  *(🖐Enumerator: if the respondent was hospitalized on more than one occasion, refer to the most important)* | Yes | | 1 | 1 |
|  |  | No | | 2  Pass to 421 | 2  Pass to 421 |
| **420** | If yes, in that admission, how many days did you stay in hospital? |  | | \|___\|___\| | \|___\|___\| |
| **…** |  | |  |  |  |
| **425ª** | 🖐 Enumerator: Could the information in chapter IVC be confirmed with a second respondent? | | Yes 1 | No 2 | NA 3 |

**Table S3 *(continued)*: Sample of questions – CONPAS 2014 and 2018**

**Panel C: Mental Health module (IV.D) – selected questions**

| 🖐*Enumerator: Read to the respondent: “The questions I will ask you now refer to certain health problems that may have bothered you in the last month (or in a typical / normal month of 2014). If you believe that you had the problem described in the last month (or in a typical / normal month of 2014), answer "YES." But if you did not have the problem in the last month (or in a typical / normal month of 2014), answer "NO".”* | | | | | |
| --- | --- | --- | --- | --- | --- |
| 🖐*Enumerator: Read to the respondent: “Now I will ask you questions referring to two different time periods: the first one being the current period, and the second one being during year 2014, when the FIFA 2014 football World Cup was held in Brazil, and the presidential election took place, electing president Juan Manuel Santos.” If the respondent is not located in the period of time (2014), refer to a positive or negative event that could have occurred to the respondent such as: a wedding, birth of a loved one, death of a loved one, etc.”.* | | | In the last month | In a typical / normal month in 2014 |  |
| **426** | Did you have frequent headaches? | Yes | 1 | 1 |  |
|  |  | No | 2 | 2 |  |
| **427** | Did you have a poor appetite? | Yes | 1 | 1 |  |
|  |  | No | 2 | 2 |  |
| **428** | Did you have trouble sleeping? | Yes | 1 | 1 |  |
|  |  | No | 2 | 2 |  |
| **…** |  |  |  |  |  |

**CONPAS internal consistency reliability**

We used standard psychometric measures to assess the internal consistency reliability of CONPAS survey modules, i.e. how well the group of question items within each of the modules measure the intended phenomena.

First, we calculated Cronbach’s alpha coefficient, which reflects the homogeneity of a single scale formed by a combination of the items in a given CONPAS module. In other words, the alpha coefficient indicates how well the many items in a single scale move together in the sample. Although there is no consensus in the literature about a minimally acceptable sample value for Cronbach’s alpha, it is common practice in applied work to consider values above 0.7 (in a scale between zero and one) to represent good reliability of the instrument for the sample of interest (Bonett and Wright 2015). Table A4 displays the Cronbach’s alpha values for the CONPAS modules examined, which are generally above 0.7.

Second, we assess CONPAS reliability further through item response theory (IRT). IRT has been argued to provide a more robust assessment of internal consistency reliability than Cronbach’s alpha, among other reasons because unlike the latter measure, IRT reliability coefficients are independent of the of items used in a given module. By contrast, Cronbach’s alpha may in principle be increased – for the same underlying individual knowledge – by varying the number of items, or even the level of difficulty of the alternative responses to an item (Nguyen et al. 2014). There is no consensus minimum threshold for IRT measures of reliability either however, for which we adopt the same 0.7 rule-of-thumb value as in other studies (Nguyen et al. 2014). Table A5 shows that IRT coefficients are generally above 0.8 for the CONPAS examined.

Although based on different testing methodologies, the results for both Cronbach’s alpha and IRT approaches indicate that the items (questions) contained within each CONPAS module complement each other well in their measurement of different aspects of the same health or wellbeing dimension of interest.

*References*

Bonett DG, Wright TA. Cronbach's alpha reliability: Interval estimation, hypothesis testing, and sample size planning. Journal of Organizational Behavior 2015; 36(1): 3-15.

Nguyen TH, Han HR, Kim MT, Chan KS. An introduction to item response theory for patient-reported outcome measurement. The Patient-Patient-Centered Outcomes Research 2014; 7(1): 23-35.

**Table S4: Cronbach’s alpha test results by CONPAS modules and survey years**

| Survey modules | Number of questions | 2019 | 2014 | Overall |
| --- | --- | --- | --- | --- |
| Household expenditures | 6 | 0.731 | 0.733 | 0.802 |
| Mental health | 25 | 0.875 | 0.864 | 0.914 |
| Health-related quality of life | 5 | 0.694 | 0.719 | 0.776 |
| Disability | 12 | 0.888 | 0.883 | 0.920 |
| Alcohol use disorder | 10 | 0.728 | 0.814 | 0.863 |
| Nicotine dependence | 6 | 0.626 | 0.658 | 0.791 |
| Selected questions across the six modules | 12 | 0.758 | 0.743 | 0.840 |

Note: We estimate Cronbach’s alpha for six core CONPAS modules that account for most of the questions of the survey (the General Health module was not included due to convergence problems to perform this test). Column 2 shows the number of questions included in each module. We report the estimated Cronbach’s alpha independently for each year, 2019 (column 3) and 2014 (column 4), and for a joint test including both years (column 5). In the last row of the table we include the following selected questions representative of each module: health expenditure; frequent headaches; trouble sleeping; feeling sad; noticed interference or something strange in their thinking; problems carrying out daily activities; difficulty taking care of household responsibilities; emotionally affected by health problems; difficulty carrying out day-to-day work or school activities; how often had a drink containing alcohol; smoked at least one cigarette per day.

**Table S5: IRT reliability test results by CONPAS modules and survey years**

| Survey modules | Number of questions | 2019 | 2014 | Overall |
| --- | --- | --- | --- | --- |
| Household expenditures | 6 | 0.998 | 1.000 | 0.962 |
| General health | 27 | 0.787 | 0.878 | 0.945 |
| Mental health | 25 | 0.889 | 0.878 | 0.931 |
| Health-related quality of life | 5 | 0.806 | 0.846 | 0.886 |
| Disability | 12 | 0.998 | 0.979 | 0.988 |
| Alcohol use disorder | 10 | 0.947 | 0.962 | 0.980 |
| Nicotine dependence | 6 | 0.562 | 0.590 | 0.771 |
| Selected questions across the seven modules | 12 | 0.902 | 0.831 | 0.902 |

Note: We perform item response theory-IRT tests for seven core CONPAS modules that account for most of the questions of the survey. Column 2 shows the number of questions included in each module. We report the estimated IRT coefficient independently for each year, 2019 (column 3) and 2014 (column 4), and for a joint test including both years (column 5). In the last row of the table we include the following selected questions representative of each module: health expenditure; self-rated general health; frequent headaches; trouble sleeping; feeling sad; noticed interference or something strange in their thinking; problems carrying out daily activities; difficulty taking care of household responsibilities; emotionally affected by health problems; difficulty carrying out day-to-day work or school activities; how often had a drink containing alcohol; smoked at least one cigarette per day.

**Table S6: Probability of attrition in the second round of CONPAS**

|  | OLS | OLS | OLS |
| --- | --- | --- | --- |
| *Conflict intensity level* |  |  |  |
| Not affected | Ref. | Ref. | Ref. |
|  |  |  |  |
| Villavicencio | 0.1353*** | 0.1145** | 0.0692 |
|  | (0.0406) | (0.0445) | (0.0439) |
| Lightly affected | 0.0345 | 0.0459 | 0.0370 |
|  | (0.0390) | (0.0464) | (0.0429) |
| Heavily affected | -0.0609** | -0.0238 | -0.0154 |
|  | (0.0271) | (0.0445) | (0.0429) |
| *Control variables* |  |  |  |
| Demographics | No | Yes | Yes |
| Population density | No | No | Yes |

Note: Demographics include displaced status, sex, age, education, employment and marital status. ** p<0.05, *** p<0.01. Taylor-linearized standard errors in parentheses account for complex survey sample design.

**Table S7: Questions selected for the test-retest approach – CONPAS 2019**

| **Question** | **Response Options** |
| --- | --- |
| Which of the following public, private or communal services did the household have [in 2014]? | 1. Electric power 2. Natural gas connected to the public grid 3. Piped water 4. Sewerage 5. Rubbish collection |
| In a typical/normal month [in 2014], how much did your household spend in total (public services, rent, food, household cleaning, clothes, etc.)? | $ (______) |
| Of the money spent [in a typical/normal month in 2014] (_______$), how much did your household spend on medicines, health supplies (orthopaedic shoes, splints, etc.), and health-related services? Include the amount for medical services (such as doctor consultations, dentist, hospital services, pharmacies) and also EPS [health insurance] co-payments/cost-sharing. But exclude the amount of what you paid or was deducted monthly for your EPS. | $ (______) |
| What was your marital status [in 2014]? | 1. Married 2. Cohabiting 3. Separated/Divorced 4. Widowed 5. Single |
| What was the highest formal education level you attained [in 2014]? (concluded) | 1. None 2. Preschool/primary 3. Secondary 4. Technical / Higher / Post-Graduate |
| How do you think your general health condition was [in 2014]? | 1. Excellent 2. Very good 3. Good 4. Fair 5. Poor |
| During 2014, were you hospitalized for any illness or another health problem (except pregnancy, but including violent events)? | 1. Yes 2. No |
| **🖐** **Enumerator:** *Read to the respondent:* *“The questions I will ask you now refer to certain health problems that may have bothered you [in a typical / normal month of 2014]. If you believe that you had the problem described [in a typical/normal month of 2014], answer "YES." But if you did not have the problem, answer "NO".”* |  |
| Did you often have headaches? | 1. Yes 2. No |
| Did you sleep badly? | 1. Yes 2. No |
| Did you notice interference or something strange in your thinking? | 1. Yes 2. No |
| In terms of your mobility, which of the following options that I am going to read to you, best describes your health in 2014? | 1. I had no problems walking 2. I had some problems walking 3. I had to stay in bed |
| "To help you describe how good or how bad your health status was in 2014, I would like you to look at a scale that is similar to a thermometer. The best state of health you can imagine should be marked with a 100 (one hundred) at the top of the scale, and the worst state of health you can imagine should be marked with a 0 (zero) at the bottom. (...show scale) Now I would like you to tell me the point on this scale where you would place your health status in 2014." | ___ |

| ***Enumerator****: In a typical/normal month in 2014, how difficult it was for you to:* |  |
| --- | --- |
| Stand for long periods of time, such as 30 minutes? | 1. Not difficult at all 2. Slightly difficult 3. Moderately difficult 4. Severely difficult 5. Extremely difficult or could not do it |
| Walk a long distance, like a kilometre [or something equivalent]? | 1. Not difficult at all 2. Slightly difficult 3. Moderately difficult 4. Severely difficult 5. Extremely difficult or could not do it |
| **Enumerator***: Read to the respondent: "Now I will ask you a few questions about your consumption of alcoholic beverages during 2014. Please try to be as honest and accurate as you can."* **Enumerator***: "Explain what you mean by "alcoholic beverages" using typical examples such as [beer, spirits, wine, vodka, etc.]. Code the responses in terms of units of consumption ("standard units")."* |  |
| How often did you consume any alcoholic beverages? | 1. Never 2. Once a month or less 3. 2 to 4 times a month 4. 2 to 3 times a week 5. 4 or more times a week |
| Did you or anyone else end up injured because you had been drinking? | 1. No   2. Yes, but before 2014  4. Yes, in 2014 |
| Did you smoke at least one cigarette per day [in 2014]? | 1. Yes 2. No |
